# Seasonal and Treatment-Related Variation in 25-Hydroxy Vitamin D Concentration in Patients with Rheumatoid Arthritis

**DOI:** 10.3390/jcm13040973

**Published:** 2024-02-08

**Authors:** Artur Cieślewicz, Katarzyna Korzeniowska, Katarzyna Grabańska-Martyńska, Anna Jabłecka, Paweł Hrycaj

**Affiliations:** 1 Department of Clinical Pharmacology, Poznan University of Medical Sciences, 61-861 Poznan, Poland; kkorzeniowska@ump.edu.pl (K.K.); ajablecka@ump.edu.pl (A.J.); 2 Department of Internal Medicine, Poznan University of Medical Sciences, 61-861 Poznan, Poland; kgrabanskamartynska@ump.edu.pl; 3 Department of Rheumatology, District Hospital in Koscian, 64-000 Koscian, Poland; pawel.hrycaj@gmail.com

**Keywords:** 25-hydroxy vitamin D, leflunomide, methotrexate, rheumatoid arthritis

## Abstract

**Background/Objectives**: 25-hydroxy vitamin D (25-OH-D) is a fat-soluble compound that plays many essential functions, including bone formation, neuromuscular functions, and prevention of osteoporosis and inflammation. Recent data indicate that its metabolites are associated with rheumatoid arthritis (RA) progression and neuropathic pain in RA patients. We aimed to assess the effect of RA pharmacotherapy and seasonal variation on serum levels of 25-OH-D in RA patients who received treatment with methotrexate (MTX) or leflunomide (LEF) for at least one year. **Methods**: This study is a retrospective analysis of data collected from 101 patients with RA who received treatment for at least one year. All of them have supplemented 25-OH-D (2000 IU daily) for at least one year. **Results**: We observed a significant seasonal variation in 25-OH-D concentration (*p* = 0.004). Moreover, there were significant differences (*p* = 0.03) between LEF (50.63 ± 17.73 ng/mL) and MTX (34.73 ± 14.04 ng/mL) treatment groups, but only for the summer population. A correlation was observed between 25-OH-D and RA duration—once again, in the summer population (the whole group—r = −0.64; treatment subgroups—r = −0.82 for LEF and −0.61 for MTX). Deficiency of 25-OH-D (below 20 ng/mL) was confirmed in 28.7% of patients, while 18.8% had suboptimal 25-OH-D levels (20–30 ng/mL). **Conclusions**: Our results showed that both RA pharmacotherapy and seasonal variation affect the serum levels of 25-OH-D in patients with active RA.

## 1. Introduction

Vitamin D3 is a fat-soluble compound that can be obtained from food (oily fish, cod liver oil, egg yolks, shiitake mushrooms, liver, or organ meats). However, its most important source is dermal synthesis after ultraviolet-B radiation [1]. Vitamin D3 synthesised by skin requires conversion to 25-hydroxy vitamin D (25-OH-D; the major circulating metabolite—a marker of vitamin D3 status and deficiency), which is then hydroxylated in the kidneys to the active form, calcitriol (1,25-dihydroxycholecalciferol; 1,25(OH)2D3) [2]. It interacts with the vitamin D receptor (VDR), which is present in many immune cell lineages, such as monocytes, dendritic cells, and activated T cells [3]. The primary physiological role of 1,25(OH)2D3 is maintaining calcium and phosphorus balance. It promotes calcium absorption in the gut and is essential for proper bone mineralisation, growth, and remodelling. Its deficiency can result in rickets and osteomalacia. Maintaining proper vitamin D3 levels protects older adults from osteoporosis [4]. Other essential functions of 1,25(OH)2D3 include reducing inflammation, modulation of cell growth, neuromuscular and immune functions, and glucose metabolism [4,5].

The worldwide data suggest that more than 50% of the population has low 25-OH-D levels [6]. A single cut-off value for 25-OH-D deficiency has yet to be established [2]. However, 20 ng/mL or higher is considered beneficial for bone condition and calcium homeostasis (with some authors stating 30 ng/mL as necessary for proper bone mineralisation) [7]. In Poland, the major factors significantly affecting 25-OH-D deficiency in the population are geographic location and climate, resulting in insufficient sun exposure during autumn and winter [8]. Recently, a great increase in 25-OH-D supplementation was noted in the Polish population due to the COVID-19 pandemic [9].

Rheumatoid arthritis (RA) is an autoimmune and inflammatory disease characterised by synovitis of the peripheral joints with extra-articular manifestations. It may result in joint destruction, loss of function, and disability and can negatively influence cardiovascular health [10]. It has recently been found that 25-OH-D level is associated with disease progression and neuropathic pain in RA patients [5,11,12].

Seasonal variations in 25-OH-D concentration are a known factor that can influence clinical manifestations of RA [13]. Methotrexate (MTX), the most common drug used in RA treatment, can also affect the 25-OH-D level by interfering with its metabolic pathways and reducing bioavailability [14]. However, studies assessing MTX’s effect on 25-OH-D concentration are limited and recruit different patient populations (oncological or juvenile idiopathic arthritis patients) [15,16]. No such data are available for leflunomide (LEF)—another drug recommended for RA treatment. Therefore, our study aims to assess the effect of RA pharmacotherapy and seasonal variation on the serum levels of 25-OH-D in patients with active RA who received treatment with methotrexate (MTX) or leflunomide (LEF) for at least one year.

## 2. Materials and Methods

This study was a retrospective analysis of data collected from 101 patients with active RA, 80 females and 21 males, aged 28–82 years, who were hospitalised at the Department of Rheumatology, Municipal Hospital, Koscian, Poland, due to exacerbation of the disease during the period of June 2021–June 2022. The inclusion criteria were as follows:

- Active RA diagnosis based on the 2010 American College of Rheumatology (ACR)—European League Against Rheumatism (EULAR) Classification Criteria for Rheumatoid Arthritis [17]: DAS28-ESR (Disease Activity Score-28 for Rheumatoid Arthritis with ESR) ≥ 2.6; DAS28-ESR was calculated on admission by the hospital’s personnel to check if the patient was in remission (score < 2.6 points) or had active disease. However, patients’ files collected for this study did not contain the score itself—only the descriptive information about the disease status (remission or active RA), which we used to include patients with active RA;
- RA treatment with MTX or LEF for at least one year;
- 25-OH-D concentration assessed;
- Supplementation with 25-OH-D (2000 IU daily) for at least one year.

The exclusion criteria were as follows:

- RA remission;
- 25-OH-D concentration not assessed;
- 25-OH-D not supplemented;
- RA treatment duration < 1 year or undetermined;
- Treatment that did not include MTX or LEF;
- Concurrent treatment with LEF and MTX;
- Gastrointestinal disorders that may affect 25-OH-D absorption;
- Ongoing oncological disease;
- Age < 18 years;
- Pregnancy.

The records were selected for patients with a high level of compliance who followed their instructions regarding clinic visits and pharmacotherapy.

Erythrocyte sedimentation rate (ESR), serum levels of 25-OH-D, thyroid-stimulating hormone (TSH), and serum C-reactive protein (CRP) were assessed during hospitalisation in the hospital’s laboratory. Patients’ demographic data (age, sex, body mass, and body mass index) and clinical data (disease duration, medications) were obtained from patient files.

We divided the included patients into the following subgroups:

1. RA treatment: LEF vs. MTX.
2. Assessment of seasonal effects:
  - The comparisons between the astronomical seasons (spring, summer, autumn, and winter);
  - The comparison between astronomical summer and the cumulative data for the remaining seasons (spring, autumn, and winter).

Statistical analysis was performed using Statistica 13.3 (TIBCO Software Inc., Palo Alto, CA, USA) and IBM SPSS Statistics 29.0.0.0 (241). A *p*-value <0.05 was considered statistically significant. The normality of distribution was evaluated using the Shapiro–Wilk test. Differences in 25-OH-D concentration between seasons were calculated using one-way ANOVA and the post hoc Tukey HSD test. Differences in 25-OH-D concentration between the two treatment groups were assessed using the *T*-test for independent samples or the Mann–Whitney U test, depending on the normality of distribution in studied groups. Correlations between variables were estimated using the Pearson correlation coefficient for normally distributed variables or Spearman’s rank correlation coefficient for variables with abnormal distribution. Multinomial logistic regression was used to estimate the effect of treatment and season on serum 25-OH-D levels.

Ethical review and approval were waived for this study due to this study being a retrospective analysis of data from medical documentation. According to the regulations of Poznan Medical University Bioethics Committee [18], ethical approval is not necessary in case of retrospective studies, e.g., including the analysis of medical records or case reports, if the results of these studies will not affect the routine management of the patient (e.g., they will not change the management during observation, e.g., as a result of conclusions obtained from the analysis).

Patient consent was waived because this study was a retrospective analysis of data from medical documentation. This paper contains no individual data that could lead to patient identification).

## 3. Results

### 3.1. Characteristics of the Studied Population

**Figure 1:**
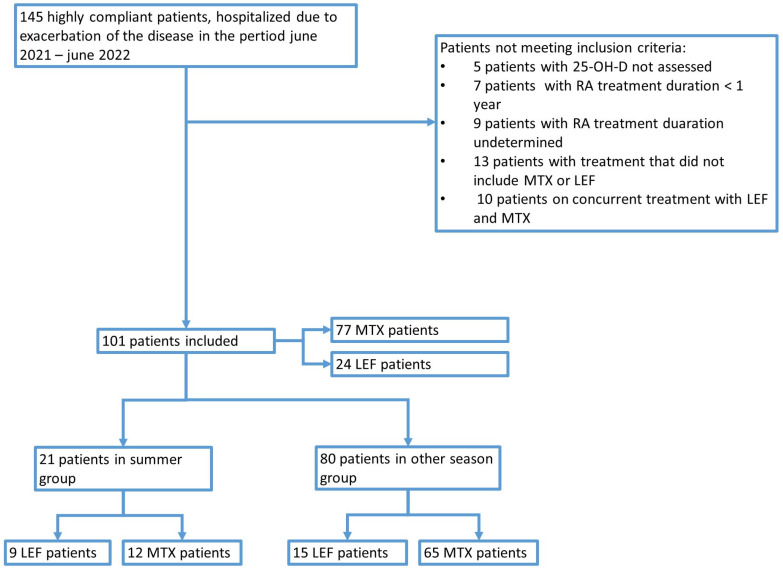
The flow diagram presenting patient recruitment for this study.

The initial number of suitable patients’ records was 145 (Figure 1). After excluding patients who did not meet the inclusion criteria, the final number included was 101. Table 1 presents the basic clinical parameters of the studied group. Mean values of 25-OH-D and TSH were within the reference range, while mean values of the body mass index (BMI), erythrocyte sedimentation rate, and CRP were above the reference range.

The largest group of patients received treatment with MTX. Concurrent treatment included such drugs as nonsteroidal anti-inflammatory drugs (NSAIDs), steroids, opioids, paracetamol, biological drugs, and Janus kinase (JAK) inhibitors (details are described in Table 2).

### 3.2. Analysis of Differences between Treatment Groups

Treatment groups did not differ significantly in age, duration of RA, body mass, BMI, ESR, CRP, TSH, and 25-OH-D levels when the whole population was analysed (Table 3).

### 3.3. Analysis of Seasonal Differences

**Figure 2:**
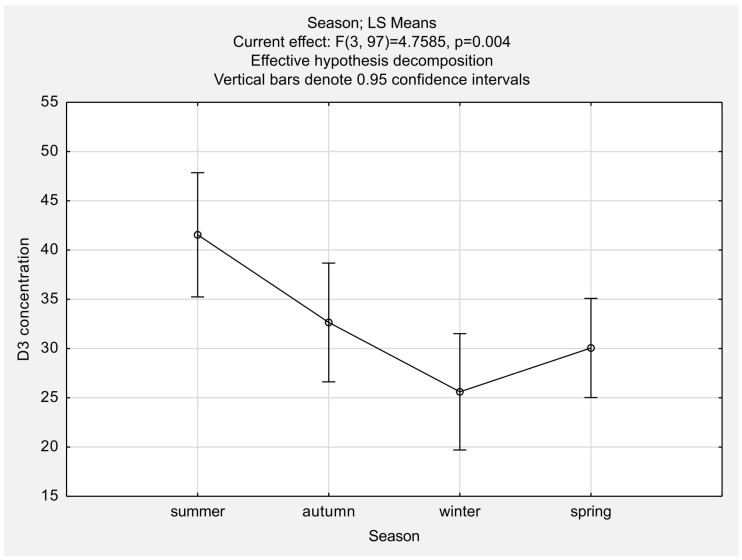
The comparison of mean serum concentrations of 25-OH-D ^1^ in different seasons. One-way ANOVA ^2^ yielded significant differences with *p* = 0.004. ^1^ 25-hydroxy vitamin D. ^2^ analysis of variance.

Statistically significant differences in 25-OH-D concentration were found depending on the measurements’ season (Figure 2).

Post hoc analysis revealed that serum 25-OH-D concentrations differed significantly in the summer group compared with the spring and the winter groups, while no significant differences existed between the spring, autumn, and winter groups (Table 4). Therefore, we decided to assess differences between the LEF and the MTX groups in two subpopulations: summer vs. the other seasons (spring + autumn + winter).

### 3.4. Analysis of Seasonal Effects in Summer vs. the Rest of the Year

**Figure 3:**
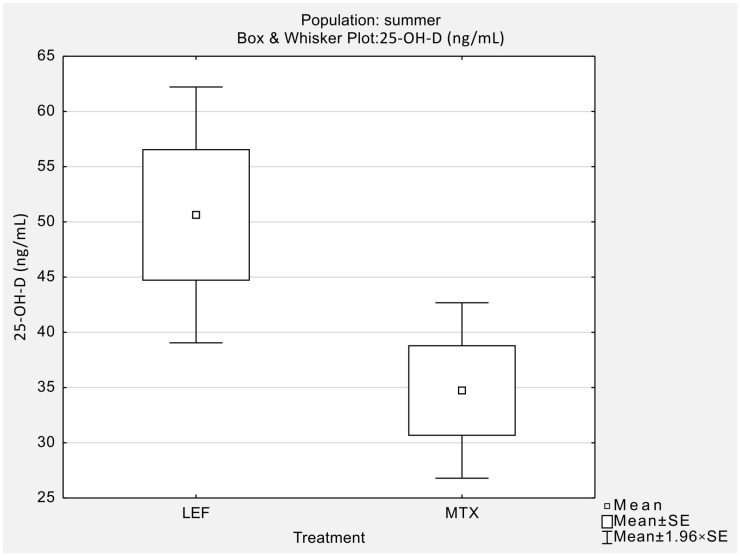
Mean 25-OH-D ^1^ concentration in LEF ^2^ and MTX ^3^ groups observed for the summer population. The difference is significant at *p* = 0.03. ^1^ 25-hydroxy vitamin D. ^2^ leflunomide. ^3^ methotrexate.

Statistical analysis revealed significant differences in serum 25-OH-D concentrations between the LEF and MTX groups in the summer population (Table 3, Figure 3). No such differences were observed in the other seasons’ population (Table 3). Moreover, 25-OH-D concentration was significantly increased during summer in the LEF group, compared to other seasons (*p* = 0.04; Table 5). No such increase was observed for the MTX group (*p* = 0.12; Table 5).

We also measured correlations between 25-OH-D concentrations and the other parameters studied. The results are presented in Table 6.

**Figure 4:**
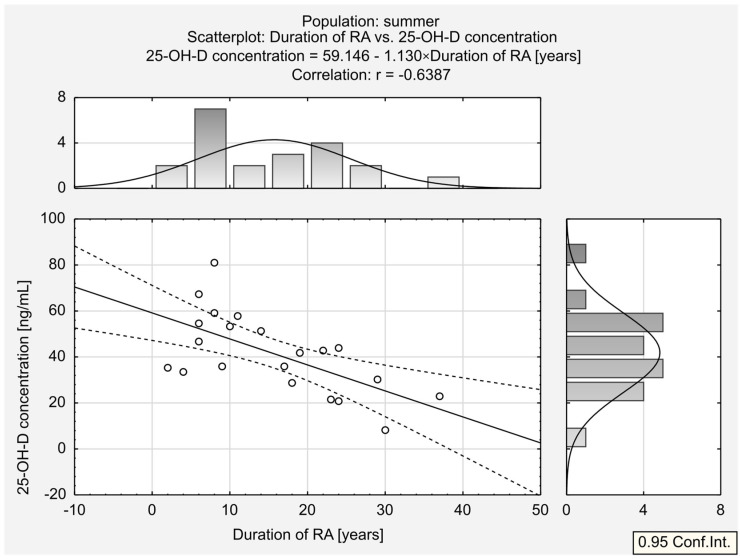
Correlation between 25-OH-D ^1^ concentration and duration of RA ^2^ in summer population. ^1^ 25-hydroxy vitamin D. ^2^ rheumatoid arthritis.

The analysis of the entire population and the two MTX and LEF treatment groups did not reveal any statistically significant correlations. A moderate correlation was found between 25-OH-D concentration and RA duration in the summer population (Figure 4), while a weak relationship was observed between 25-OH-D concentration and the body mass in the other seasons’ population.

**Figure 5:**
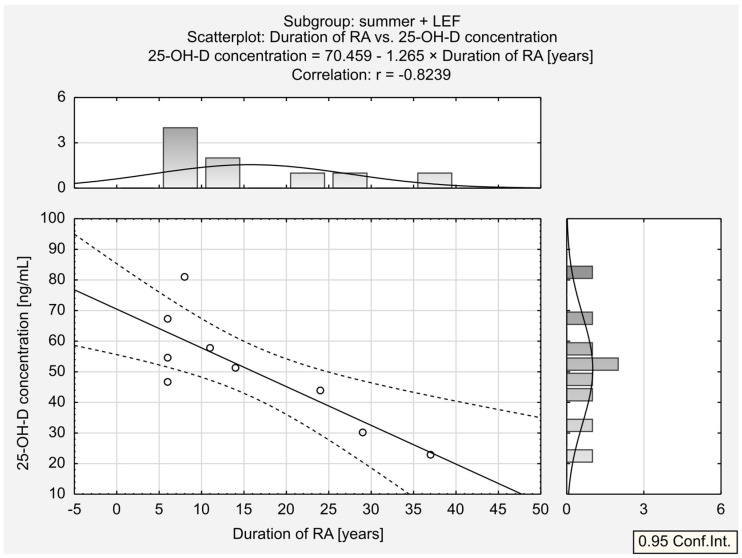
Correlation between 25-OH-D ^1^ concentration and duration of RA ^2^ in summer + LEF ^3^ subgroup. ^1^ 25-hydroxy vitamin D. ^2^ rheumatoid arthritis. *^3^* leflunomide.

**Figure 6:**
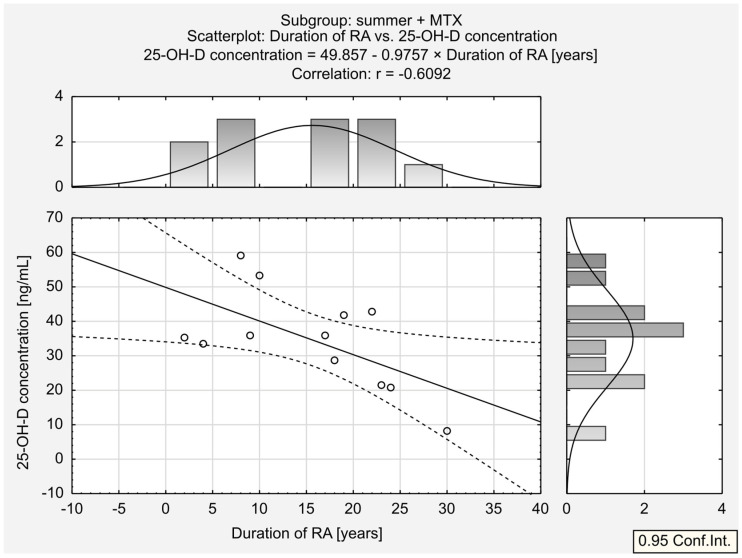
Correlation between 25-OH-D ^1^ concentration and duration of RA ^2^ in summer + MTX ^3^ subgroup.^1^ 25-hydroxy vitamin D. ^2^ rheumatoid arthritis. ^3^ methotrexate.

We then divided the two seasonal populations into subgroups depending on the treatment. A substantial positive relationship was observed between serum 25-OH-D concentration and ESR in the summer + LEF group. A similar trend was found for the other seasons + MTX group; however, its magnitude was low this time. A substantial negative correlation was observed between 25-OH-D concentration and the body mass in the other seasons + LEF group. Moreover, a moderate negative correlation was observed between 25-OH-D concentration and BMI in the other seasons + LEF group. Interestingly, the moderate correlation between 25-OH-D concentration and RA duration observed in the summer group was also found in the summer + LEF (strong relationship; Figure 5) and the summer + MTX groups (substantial relationship; Figure 6).

### 3.5. Analysis of 25-OH-D Deficiency in the Studied Population

Based on the measured 25-OH-D concentration, patients were categorised as follows:

- Deficient (<20 ng/mL);
- Suboptimal (20–30 ng/mL);
- Correct (30–50 ng/mL);
- Increased (>50 ng/mL).

Table 7 presents patients fitting into each category for the studied population and in the LEF, MTX, summer, and other seasons’ groups.

In almost 30% of patients, 25-OH-D deficiency was present, while nearly 50% had a 25-OH-D concentration below 30 ng/mL. Multinomial logistic regression analysis showed that both treatment and season were statistically significant (Table 7). The chance for 25-OH-D correct or increased levels was significantly lower, compared to deficiency, in the other seasons’ group (Table 8). Moreover, patients treated with LEF had a significantly higher chance for increased 25-OH-D levels, compared to suboptimal and correct, than patients from the MTX group (Table 8).

## 4. Discussion

In our study, we wanted to evaluate the potential influence of RA treatment (LEF or MTX) and seasonal variation on the serum levels of 25-OH-D. We found a significantly elevated 25-OH-D concentration in patients who had it measured during the summer season. Moreover, the effect of treatment on 25-OH-D level was noticeable only in the summer group, where patients treated with LEF had significantly higher 25-OH-D concentrations than patients on MET treatment. Interestingly, while a significant increase in 25-OH-D level was confirmed in the summer group of LEF-treated patients compared to the other seasons’ group, no such increase was found for MET patients. Additionally, we observed a negative correlation between 25-OH-D concentration and RA duration, but once again, only for the summer group. We also analysed the 25-OH-D deficiency status in the studied population, confirming the presence of deficiency in almost 30% of patients, while a suboptimal level was observed in nearly 50% of patients. It was affected by both treatment and season.

Besides maintaining calcium homeostasis, 25-OH-D has substantial immunomodulatory activity. Its anti-inflammatory mechanisms include the inhibition of prostaglandin synthesis, the nuclear translocation of NF-kB, stress-activated kinase signalling, and the production of inflammatory cytokines [12]. An increasing amount of data suggests the association between 25-OH-D insufficiency/deficiency and various musculoskeletal and autoimmune disorders, including RA [19]. A prospective study on 29,368 women aged 55–69 without a history of RA revealed that greater vitamin D3 intake was inversely associated with the risk of developing RA during 11 years of follow-up [20]. Kostoglou-Athanassiou et al. observed a decreased 25-OH-D concentration in a cohort of 44 RA patients compared to healthy controls. Moreover, vitamin D3 levels were negatively correlated with the disease activity score index of a 28-joint count (DAS28) [21]. A meta-analysis by Song et al., including three cohort studies with 215,757 participants (874 RA cases) and eight studies on 2,885 RA patients and 1,084 controls, found an association between vitamin D3 intake and RA incidence. In 10 studies, the correlation between vitamin D3 levels and disease activity was confirmed [22]. Sharma et al. observed a high prevalence of vitamin D3 deficiency and insufficiency in 80 RA patients. Moreover, they found a negative correlation between 25-OH-D concentration and disease activity [23]. The TOMORROW study, including 208 RA patients and 205 controls, revealed significantly lower 25-OH-D levels in RA patients, although there was no correlation between vitamin D3 concentration and disease activity [24]. On the contrary, Azzeh et al., in their study on 102 RA patients, found a significant negative correlation between serum 25-OH-D level and DAS28 [25]. Lin et al. conducted a meta-analysis of 24 studies recruiting 3489 RA patients. They found lower vitamin D3 levels in RA patients than healthy controls and a negative relationship between 25-OH-D concentration and disease activity index [12]. A meta-analysis by Lee and Bae yielded similar results, including 15 studies on 1143 RA patients and 963 controls. It confirmed the lowered serum 25-OH-D concentration and higher prevalence of vitamin D3 deficiency in RA patients and showed a significant negative correlation between the 25-OH-D level and disease activity [26]. Similar conclusions were reached by the first multicentre European survey to assess vitamin D3 status in RA patients. The international study recruited 625 RA patients and 276 age- and sex-matched healthy subjects and revealed significantly lower mean 25-OH-D concentrations in RA patients and a negative correlation between vitamin D3 level and disease activity [27]. The authors involved in the COMORA research study, recruiting 1413 RA patients from 15 countries, observed a high prevalence of vitamin D3 insufficiency and deficiency among RA patients and found an association between low 25-OH-D level and disease activity [28]. Mateen et al., in their study on 100 RA patients and 50 healthy controls, found decreased 25-OH-D concentrations in the RA group. Moreover, they observed increased inflammatory cytokines and reactive oxygen species (ROS) levels in RA patients compared to the healthy controls. Notably, cytokine levels were negatively correlated with 25-OHD and positively with ROS [29]. A study on 150 RA patients treated with disease-modifying antirheumatic drugs (DMARDs) revealed that supplementation with vitamin D3 could significantly improve disease activity [30]. Similar results were achieved by Mukherjee et al.—patients supplemented with 60,000 IU of 1,25(OH)2D3 reported significantly higher median pain relief scores and DAS28 scores [31]. Liu and Wen, in their study on 280 treatment-naive RA patients and 140 healthy controls, observed a significant association between vitamin D3 and DAS28 score [32]. According to an Italian study on 61 RA patients who received 100,000 IU of vitamin D3, the effectiveness of the supplementation varied depending on the baseline status of 25-OH-D. In the case of patients without vitamin D3 deficiency, the supplementation was associated with an improvement in DAS28-CRP, while no such improvement was observed in patients with vitamin D3 deficiency; however, a significant improvement in pain was observed for the latter group [33]. A recent meta-analysis of six studies, with a total of 438 participants, revealed that vitamin D3 supplementation of RA patients led to a significant improvement in DAS28, erythrocyte sedimentation rate (ESR), and tender joint count (TJC) [34]. Mouterde et al. conducted a multicentre study on 645 RA patients from 14 French rheumatology centres to evaluate the association between vitamin D3 concentration and disease activity. A deficiency and insufficiency in 25-OH-D were observed in 82% of patients. Moreover, vitamin D3 deficiency was associated with increased severity of the disease [35]. Summarising, a lot of data indicate the association between RA and 25-OH-D concentration.

Seasonal variations in 25-OH-D concentration depend on geographic location and is a factor that can lead to seasonal differences in RA activity [13,36,37,38]. A study on the Swedish population revealed a significant seasonal variation in 25-OH-D concentration associated with the intensity of UV-B irradiation [36]. Herly et al. conducted a study on the Danish population and concluded that both vitamin D level and season were associated with achieving a one-year remission of newly diagnosed RA [38]. On the other hand, studies recruiting patients from regions with greater sun exposure did not confirm seasonal variations in 25-OH-D levels or correlations between 25-OH-D and RA activity [37,39]. For Poland, a seasonal variation in 25-OH-D concentration was confirmed in adult, adolescent, and child populations [7,40]. Our observations are consistent with these results—a seasonal variation was noted, with the highest 25-OH-D level in summer and the lowest in winter.

The pharmacotherapy of RA relies on using conventional synthetic DMARDs, such as MTX (first-line treatment), LEF or sulfasalazine (in case of MTX intolerance or contraindications), biological DMARDs, targeted synthetic DMARDs, glucocorticoids, NSAIDs, and other analgesics [41].

MTX is the main DMARD of RA treatment, mainly because of the low price and good treatment response [42]. It is a folate antagonist with anti-proliferative, anti-metabolic, and anti-inflammatory effects [42]. MTX inhibits dihydrofolate reductase activity, leading to tetrahydrofolate deficiency, thus disturbing the synthesis of purines and pyrimidines. As a result, deoxyribonucleic acid (DNA), ribonucleic acid (RNA), and protein biosynthesis are inhibited, which prevents the proliferation of inflammatory cells responsible for RA [42]. Other mechanisms involved in the MTX effect on RA include the anti-inflammatory effect via the adenosine signalling pathway, regulation of the receptor activator for nuclear factor κ B ligand (RANKL)/receptor activator for nuclear factor κ B (RANK)/osteoprotegerin (OPG) pathway, and regulation of the number of various T cells (the reduction in Th1 and Th17 cell proportion with the increase in Th2 and regulatory T (Treg) cells) [42].

LEF is an oral prodrug rapidly metabolised to its active metabolite A77 1726 (teriflunomide). Its mechanism of action involves the inhibition of a mitochondrial enzyme dihydroorotate dehydrogenase, preventing de novo pyrimidine ribonucleotide uridine monophosphate (rUMP) synthesis. It leads to decreased DNA and RNA synthesis, cell proliferation inhibition, and cell retention in the G1 phase. As a result, LEF blocks the proliferation of autoimmune T cells and provides anti-inflammatory and immunomodulatory effects [43]. LEF is a recommended DMARD for RA in combination with MTX or as a monotherapy for MTX nonresponders [44]. Other disorders that can benefit from LEF treatment include psoriatic arthritis, antineutrophil cytoplasmic autoantibody-associated vasculitis, SLE, and Takayasu disease [45].

Most of our patients were treated with MTX. We found a difference in 25-OH-D concentration between patients treated with MTX and LEF; however, the difference was statistically significant only in the summer population. In our opinion, this observation is quite valuable, as no other studies have been published that evaluate treatment and seasons as factors that can influence 25-OH-D concentration in RA patients. We found the closest reference to our results in a cross-sectional study by Stawicki et al., conducted on 189 Caucasian children and adolescents with juvenile idiopathic arthritis (JIA) [16]. They observed that MTX treatment decreased 25-OH-D concentration and found an inverse relationship between the dose of MTX and 25-OH-D level. The mechanism of MTX interference with vitamin D3 metabolism is unclear. The authors considered such hypotheses as the deterioration of vitamin D3 bioavailability from nutrients, the downregulation of the hepatic hydroxylation of calciferol (due to the drug hepatotoxicity), and the disturbance of vitamin D3 skin synthesis [16]. A recent paper published by Su et al. presents some interesting data concerning the effect of MTX chemotherapy on 25-OH-D levels in an animal model [14]. The authors confirmed that MTX treatment led to a significant decrease in 25-OH-D serum concentration. Moreover, they found that MTX chemotherapy affected the intestinal expression of enzymes crucial in vitamin D metabolism, resulting in an increased expression of CYP27B1 (responsible for converting 25-OH-D to active form) and then CYP24 (vitamin D catabolism). They also observed intestinal damage induced by MTX treatment, which can negatively influence vitamin D absorption from nutrients [14]. We observed a significantly decreased level of 25-OH-D in the summer group treated with MTX, compared with LEF-treated patients. Moreover, we did not find any significant increase in 25-OH-D concentration in the MTX summer group compared with the other seasons’ group, while such an increase did occur for LEF-treated patients. It led us to hypothesise that MTX could interfere with the vitamin D3 skin biosynthesis process. The mechanism described by Su et al., involving increased expressions of CYP27B1 and CYP24 and decreased intestinal absorption, could play a role here. However, it should be underlined that results from animal studies can differ from those observed in humans; moreover, the authors used an animal model of MTX treatment used in oncology, which is different from RA therapy. Another consideration is that MTX potentially causes photosensitivity reactions, which may lead to limited sun exposure and decreased 25-OH-D biosynthesis [46]. Additional studies are needed to provide more insight.

An interesting observation found in our population was the negative correlation between RA duration and 25-OH-D concentration in the summer population. One of the potential explanations, consistent with our results, includes the high prevalence of vitamin D3 deficiency among RA patients and its association with disease progression and severity [12,19,20,21,22,23,24,25,26,27,28,29,40]. Another factor worth considering is that the skin decreases its capacity to produce previtamin D3 with age (approximately 13% per decade); however, we did not observe any correlations between age and D3 concentration [47].

A deficiency in 25-OH-D is prevalent in the Polish population. According to Płudowski et al., the prevalence of 25-OH-D concentrations below 30 ng/mL was 89.9% out of 5775 adult volunteers from 22 Polish cities, with 65.8% with a deficient level (less than 20 ng/mL) and 16% experiencing severe hypovitaminosis (25-OH-D level below 10 ng/mL) [7]. Similar data were presented in the work of Kmieć et al.—81.1% of primarily young and middle-aged participants had 25-OH-D deficiency during the winter season, suggesting that ultraviolet (UV) exposure during the summer was insufficient to maintain correct vitamin D3 levels [48]. Interestingly, insufficient levels of 25-OH-D were less frequent in our population (18.8% with 25-OH-D concentration between 20 and 30 ng/mL; 28.7% with a deficient concentration of less than 20 ng/mL). The most probable explanation for this difference is supplementation with 25-OH-D, which all patients in our study reported. Such routine supplementation is less frequently noted in the healthy population. According to the study of Waszak et al., conducted on 1766 student volunteers, up to 44% of the participants were taking at least one vitamin D3 supplement [49]. Similar results were delivered by a cross-sectional study among 595 medical university students: 50.9% of the respondents declared supplementation of vitamin D3 [50].

The present study has several limitations that should be considered when interpreting the results. Firstly, the sample size was relatively small, especially when it was divided into season and treatment groups. It was particularly severe for LEF-treated patients, who constituted the minority of the whole studied population. A larger sample size would have provided more statistical power and allowed for more robust conclusions. Secondly, there was an unequal distribution of patients between the two treatment groups (LEF and MTX), which resulted from MTX being the primary DMARD used in the treatment of RA and may introduce bias and confounding factors. Although the two treatment subgroups in the summer population were comparable, their small size was a significant limiting factor. Lastly, an essential limitation of this study was the lack of DAS28 assessment, which makes it difficult to compare our results with other studies. Therefore, caution should be exercised when interpreting the results of this study, and further research with larger sample sizes should be conducted to address these limitations and enhance the validity of the findings.

On the other hand, we would like to emphasise that although there is research published assessing 25-OH-D in RA patients, our study seems to be the first evaluating the effect of treatment (MTX vs. LEF) and seasonal variation, thus adding new valuable insight [51,52,53]. Our results indicated the difference in 25-OH-D levels between LEF and MTX patients that was noticeable only during the summer season. We plan to confirm this observation on a larger sample of patients (especially considering the lower number of patients on LEF treatment). The increased number of patients would also enable us to assess different treatment options (e.g., biological drugs), which we had to exclude due to a very small sample size. We also plan to collect some survey data from the patients (e.g., concerning the exposure to sunlight or usage of UV-protection creams). Finally, it would be worth carrying out an in vitro study (for example, using HaCaT cell line [54]) to assess if exposure to MTX can directly decrease light-induced vitamin D biosynthesis.

## 5. Conclusions

Our results showed a possible link between 25-OH-D concentration and treatment with MTX. Such an observation has been reported only in one study on human subjects and one animal model. A possible mechanism may be associated with MTX interfering with the process of vitamin D3 light-induced biosynthesis and metabolism, involving the increased expression of CYP27B1 and CYP24 enzymes. Other factors that should be considered are the decreased vitamin D absorption due to MTX-induced intestinal damage and a possible decrease in sun exposure because of potential photosensitivity reactions associated with MTX treatment. However, further research on a larger population is required to provide more insight into this finding. It is also worth underlining that supplementation with 25-OH-D may significantly decrease the frequency of deficiency (which we observed in our study), therefore having a beneficial impact on the progression of RA.

## Figures and Tables

**Table 1:** Basic clinical characteristics of patients with RA ^1^.

Parameter [Unit] [Reference Range]	Valid N	Mean ± SD	Median	Minimum	Maximum
Age [years]	101	60 ± 11	62	28	82
Duration of RA [years]	101	12 ± 10	8	1	41
Body mass [kg]	101	77.47 ± 18.06	73.80	49.00	133.80
BMI [kg/m^2^] [18.5–24.9]	101	29.55 ± 7.13	28.00	18.70	53.40
ESR [mm/one hour] [3–15]	101	21.80 ± 17.24	17.00	2.00	94.00
CRP [mg/L] [0.0–5.0]	101	10.17 ± 15.43	4.00	0.00	87.50
25-OH-D [ng/mL] [30–100]	101	31.98 ± 15.37	31.40	8.20	81.00
TSH [uIU/mL] [0.35–4.94]	99	1.79 ± 2.23	1.27	0.13	17.24

^1^ rheumatoid arthritis.

**Table 2:** Pharmacotherapy of patients with RA ^1^.

	Whole Population (*n* = 101)	MTX (n = 77)	LEF (*n* = 24)
Paracetamol	23	19	4
NSAIDS ^2^	60	44	16
Opioids ^3^	28	22	6
Steroids ^4^	37	28	9
Biological ^5^	9	8	1
Janus kinase (JAK) inhibitors ^6^	3	3	0

^1^ rheumatoid arthritis. ^2^ aceclofenac, acemetacin, celecoxib, dexketoprofen, diclofenac, etoricoxib, lornoxicam, meloxicam, naproxen. ^3^ codeine, naloxone, oxycodone, tramadol. ^4^ methylprednisolone, prednisone. ^5^ adalimumab, etanercept, golimumab, rituximab, tocilizumab. ^6^ baricitinib, tofacitinib.

**Table 3:** Differences in the studied parameters between the LEF ^1^ and the MTX ^2^ groups for the whole studied population and in summer and other seasons’ populations.

Variable	Valid N in LEF Group	Valid N in MTX Group	Mean (SD) Value in LEF Group	Mean (SD) Value in MTX Group	Median in LEF Group	Median in MTX Group	*p*
Whole population							
Age [year]	24	77	60 (13)	60 (11)	62	62	0.85
Duration of RA ^3^ [years]	24	77	11 (10)	13 (10)	6	9	0.43
Body mass [kg]	24	77	73.38 (19.03)	78.74 (19.03)	69.30	74.60	0.35
BMI ^4^ [kg/m^2^]	24	77	28.91 (7.43)	29.75 (7.43)	28.85	27.90	0.80
ESR ^5^ [mm/one hour]	24	77	24.54 (15.65)	20.95 (15.65)	17.50	17.00	0.63
CRP ^6^ [mg/L]	24	77	12.01 (13.46)	9.60 (13.46)	1.65	4.80	0.17
25-OH-D ^7^ [ng/mL]	24	77	39.41 (12.38)	29.66 (12.38)	38.30	29.00	0.07
TSH ^8^ [uIU/mL]	23	76	1.88 (2.15)	1.76 (2.15)	1.27	1.24	0.76
Summer population							
Age [years]	9	12	65 (7)	63 (9)	66	64	0.59
Duration of RA [years]	9	12	16 (12)	16 (9)	11	18	0.97
Body mass [kg]	9	12	74.69 (11.58)	83.38 (23.96)	74.00	77.75	0.78
BMI [kg/m^2^]	9	12	30.87 (5.64)	33.21 (10.16)	29.30	29.80	0.75
ESR [mm/one hour]	9	12	27.00 (28.08)	23.75 (13.41)	17.00	21.50	0.72
CRP [mg/L]	9	12	15.76 (26.02)	12.89 (15.16)	1.70	5.95	0.46
25-OH-D [ng/mL]	9	12	50.63 (17.73)	34.73 (14.04)	51.30	35.60	0.03
TSH [uIU/mL]	9	12	2.84 (3.89)	2.81 (4.68)	1.37	1.35	0.97
Other seasons’ population							
Age [year]	15	65	57 (15)	60 (12)	58	62	0.41
Duration of RA [years]	15	65	9 (9)	12 (10)	5	8	0.12
Body mass [kg]	15	65	72.60 (15.75)	77.89 (18.08)	67.80	74.60	0.36
BMI [kg/m^2^]	15	65	27.74 (6.31)	29.12 (6.72)	26.00	27.00	0.43
ESR [mm/one hour]	15	65	23.07 (17.85)	20.43 (16.07)	18.00	17.00	0.55
CRP [mg/L]	15	65	9.77 (17.55)	8.99 (13.16)	1.60	4.40	0.22
25-OH-D [ng/mL]	15	65	32.67 (20.55)	28.72 (11.93)	30.10	27.50	0.99
TSH [uIU/mL]	14	64	1.26 (0.81)	1.56 (1.19)	1.07	1.24	0.60

^1^ leflunomide. ^2^ methotrexate. ^3^ rheumatoid arthritis. ^4^ body mass index. ^5^ erythrocyte sedimentation rate. ^6^ C-reactive protein. ^7^ 25-hydroxy vitamin D. ^8^ thyroid-stimulating hormone.

**Table 4:** Results of post hoc analysis of differences between season groups using Tukey’s HSD ^1^ test.

Season	Summer	Autumn	Winter
Autumn	0.19		
Winter	0.002	0.35	
Spring	0.03	0.91	0.67

^1^ honestly significant difference.

**Table 5:** Differences in 25-OH-D ^1^ concentration during summer and other seasons for LEF ^2^ and MTX ^3^ groups.

Group	Valid N in Summer Group	Valid N in Other Seasons Group	Mean 25-OH-D Concentration in Summer Group	Mean 25-OH-D Concentration in Other Seasons Group	Median in Summer Group	Median in Other Seasons Group	*p*
LEF	9	15	50.63 ± 17.73	32.67 ± 20.55	51.30	30.10	0.04
MTX	12	65	34.73 ± 14.04	28.72 ± 11.93	35.60	27.50	0.12

^1^ 25-hydroxy vitamin D. ^2^ leflunomide. ^3^ methotrexate.

**Table 6:** Correlation between serum 25-OH-D ^1^ concentrations and age, duration of RA ^2^, body mass, TSH ^3^ concentration, BMI ^4^, ESR ^5^, and CRP ^6^ level assessed for the whole population, seasonal, and seasonal + treatment subgroups. Statistically significant correlations indicated with * (*p* < 0.05).

	Whole Population	LEF ^7^	MTX ^8^	Summer	Other Seasons	Summer + LEF	Summer + MTX	Other Seasons + LEF	Other Seasons + MTX
Age [years]	0.17	0.21	0.16	0.09	0.15	0.00	0.07	0.09	0.16
Duration of RA [years]	0.08	−0.02	0.13	−0.64 *	0.14	−0.82 *	−0.61 *	−0.19	0.18
Body mass [kg]	−0.11	−0.33	0.01	0.25	−0.22 *	0.17	0.47	−0.66 *	−0.10
BMI [kg/m^2^]	−0.03	−0.11	0.05	0.28	−0.17	0.31	0.50	−0.57 *	−0.06
ESR [mm/one hour]	0.17	0.22	0.20	0.03	0.18	0.67 *	−0.45	−0.03	0.26 *
CRP [mg/L]	0.01	−0.06	0.10	−0.21	0.05	−0.03	−0.43	−0.30	0.17
TSH [uIU/mL]	−0.02	0.06	−0.01	0.15	−0.06	0.18	0.16	−0.04	−0.04

^1^ 25-hydroxy vitamin D. ^2^ rheumatoid arthritis. ^3^ thyroid-stimulating hormone. ^4^ body mass index. ^5^ erythrocyte sedimentation rate. ^6^ C-reactive protein. ^7^ leflunomide. ^8^ methotrexate.

**Table 7:** Characteristics of 25-OH-D ^1^ levels in the studied population. Values are presented as counts (percentage).

	Deficient	Suboptimal	Correct	Increased	Total	Likelihood Ratio *p*
whole population	29 (28.7)	19 (18.8)	40 (39.6)	13 (12.9)	101	
LEF	7 (29.2)	1 (4.2)	7 (29.2)	9 (37.5)	24	0.002
MTX	22 (28.6)	18 (23.4)	33 (42.9)	4 (5.2)	77	
summer	1 (4.8)	4 (19.0)	9 (42.9)	7 (33.3)	21	0.009
other seasons	28 (35.0)	15 (18.8)	31 (38.8)	6 (7.5)	80	

^1^ 25-hydroxy vitamin D.

**Table 8:** Odds ratio (OR) values calculated for the 25-OH-D ^1^ level. The 95% confidence interval is presented in square brackets. Statistical significance is marked with * (*p* < 0.05).

			Reference Category		
			Deficiency	Suboptimal	Correct
Estimated categories	suboptimal	treatment = LEF	0.138 [0.015–1.291]		
		season = other seasons	0.104 [0.010–1.071]		
	correct	treatment = LEF	0.531 [0.153–1.844]	3.858 [0.432–34.420]	
		season = other seasons	0.110 * [0.013–0.943]	1.055 [0.271–4.103]	
	increased	treatment = LEF	4.596 [0.962–21.964]	33.389 * [3.156–353.218]	8.655 * [1.989–37.654]
		season = other seasons	0.043 * [0.004–0.437]	0.415 [0.071–2.415]	0.393 [0.091–1.702]

^1^ 25-hydroxy vitamin D.

## Data Availability

The data presented in this study are available on request from the corresponding author. The data are not publicly available, due to privacy restrictions.
